# Case report: Emergency presentation of Meckel's diverticulum in the 3rd trimester of pregnancy

**DOI:** 10.3389/fsurg.2023.1051158

**Published:** 2023-02-16

**Authors:** Yantao He, Lilan Wang, Wulan Cao

**Affiliations:** Department of Obstetrics, Zhongshan City People's Hospital, Zhongshan, China

**Keywords:** meckel's diverticulum, acute abdomen, pregnancy, emergency, obstetric (third trimester)

## Abstract

**Background:**

Symptomatic Meckel's diverticulum (MD) is easily neglected in the acute abdomen during pregnancy. MD is the most common congenitally anomalous development of the intestines, with an incidence of 2% in the general population, although it is not easily diagnosed because of variable clinical features. Especially when complicated with pregnancy, doctors can easily overlook this disease, which directly threatens maternal and foetal life.

**Case Presentation:**

We report the case of a 25-year-old at 32 + 2 weeks of gestation complicated with MD volvulus who presented with progressive abdominal pain and finally peritonitis. She underwent exploratory laparotomy and small-bowel resection. The mother and the baby successfully recovered.

**Conclusions:**

MD-complicated pregnancy is not easily diagnosed. Once highly suspiciously diagnosed, especially with peritonitis, surgery should be arranged, which helps preserve maternal and foetal life.

## Introduction

MD is the most common congenital anomaly of the gastrointestinal tract, caused by incomplete obliteration of the omphalomesenteric duct in the developing embryo, which has an incidence of 2% (1, 2). Patients with MD are usually asymptomatic, while 3.7%–6.4% present various symptoms, such as small-bowel obstruction, peritonitis, appendicitis, cholecystitis, renal colic disease, or peptic ulcer disease (3, 4). MD is easily misdiagnosed because the clinical features vary between individuals. In particular, MD complicates late pregnancy, and the diagnosis can be delayed, leading to high mortality of the mother and foetus.

Here, we report a young woman at 32 + 2 weeks of gestation complicated with MD volvulus who presented with progressive abdominal pain and finally peritonitis. Meckel's diverticulum is the third trimester of pregnancy is rare. We summarized five such cases that we found in the existing literature in Table 1.

**Table 1:** Cases of Meckel's diverticulum in the third trimester of pregnancy.

Age (years)	Gestational age (weeks)	Symptoms	Peritonitis	Imaging	Treatment	Result	Apgar scores (1,5 min)
34	34	Abdominal pain	+	Abdominal x-ray	Surgery	Mother and baby recovered	6,9 (5)
14	32	Abdominal pain, distension	+	CT	Surgery, the patient was discharged. Spontaneous labor and vaginal delivery at term	Mother and baby were healthy	Unknown (6)
40	33	Abdominal pain, nausea, vomiting	+	CT	Surgery	Mother and baby recovered	9,10 (7)
30	37	Abdominal pain	+	CT	Surgeryand eight hours postoperatively preterm labor	Mother recovered and the baby in the neonatal intensive care unit	0,2 (8)
23	29	Abdominal pain, nausea, vomiting	+	CT	Surgery, the patient was discharged.cesarean section at 36 weeks	Mother and baby recovered	Unknown (9)

## Case presentation

A 25-year-old at 32 + 2 weeks gestation arrived at the hospital because of abdominal pain with a recent ingestion of spicy food. Approximately 4 h later, she felt abdominal colic pain, which improved spontaneously or by changing position, with vomiting 5 times. Without diarrhoea, she defecated normal soft stool twice. She had no fever. The pain was not relieved after she received the phloroglucinol at a dose of 40 mg iv in the outpatient department. Then, she was transferred to the inpatient department for treatment at 17:00.

Physical examination showed a pregnant abdomen, which was consistent with the gestational age. The patient had total abdominal pain, mainly in the lower abdomen, with tenderness and rebound pain. She had no noticeable uterine contractions, pain, or feelings of tightness. The cervix was closed.

Ultrasound testing of the foetus and the placenta was normal. Antepartum testing was also normal. Foetal surveillance was normal, and there were no obvious uterine contractions.

The patient was not considered to be in preterm labour. The cause of her abdominal pain was investigated, and acute gastroenteritis was considered. When she arrived at our inpatient department, we did not use dexamethasone considering that there was no uterine contraction pain.

**Figure 1:**
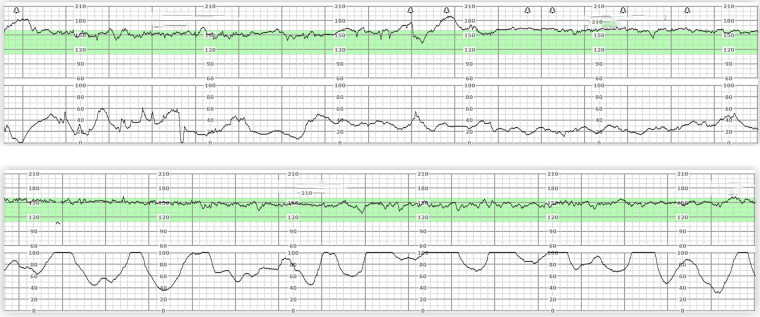
Electronic fetal monitor on admission and fetal monitor with contractions.

Four hours after admission (at approximately 21:30), the patient felt increased pain in her left lower quadrant. She had total abdominal pain, mainly in the left lower abdomen, with tenderness and rebound pain. Strong, regular contractions were occurring every 3 min (Figure 1), and we started medication to prolong the period of gestation as soon as possible. Dexamethasone was administered to enhance foetal lung maturity, and intravenous tocolysis with ritodrine was started. Intravenous cefuroxime sodium was used to prevent infection.

**Figure 2:**
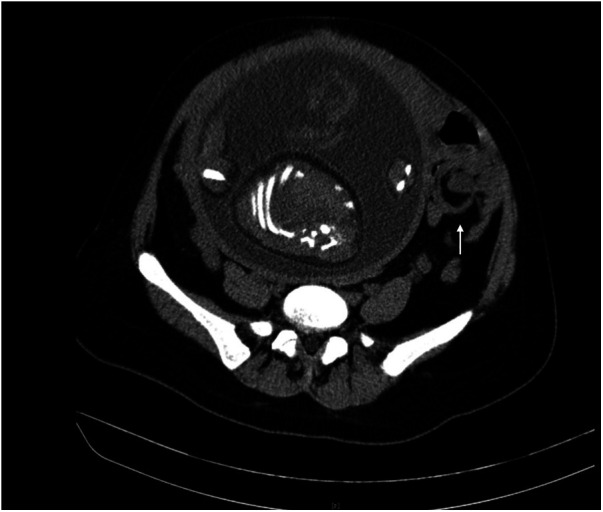
The left lower abdominal part of the small intestine was in a vortex shape in CT.

The white blood cell count was 16,530, and the percentage of neutrophils was 86.20%. Procalcitonin, interleukin-6, C-reactive protein, pancreatic amylase, and pancreatic lipase levels were normal. As the patient's abdominal pain worsened, we examined the routine blood and infection indicators again. The white blood cell count was 20,670, and the percentage of neutrophils was 90.30%. Procalcitonin was 0.03 ng/ml, interleukin-6 was 44.90 pg/ml, and C-reactive protein was 7.90 mg/L. Computed tomography (CT) examinations are associated with ionizing radiation and are not advised in pregnancy, which could have a negative effect on the foetus, mainly depending on the the absorbed radiation dose and the gestational age at the time of exposure. However, CT examination may improve the diagnosis accuracy in the acute abdomen, especially intestinal obstruction and appendicitis. The patient was informed of the advantages and disadvantages of CT examinations. With the patient's permission, we immediately contacted the imaging department to perform B-ultrasound and CT examination. CT examination showed that the left lower abdominal part of the small intestine was in a vortex shape, and the local intestinal lumen was dilated. The intestinal inflammation was distinguished from the incomplete small intestinal torsion (Figure 2). No obvious abnormalities were found in the liver, gallbladder, spleen, pancreas or ureter.

The next morning, the patient continued to feel abdominal pain, and she presented obvious tenderness and rebound pain, which showed peritonitis. We consulted radiologist and surgeon and they considered that the patient could have volvulus. The surgeon suggested that exploratory laparotomy be performed. Due to the large size of the pregnant uterus, which would obstruct the surgical field of view, combined with the gestational stage, the surgeon recommended emergency caesarean section in obstetrics, and the surgeon would assist in the exploration. The complete 10 mg dexamethasone injection was empirically administered before the operation.

**Figure 3:**
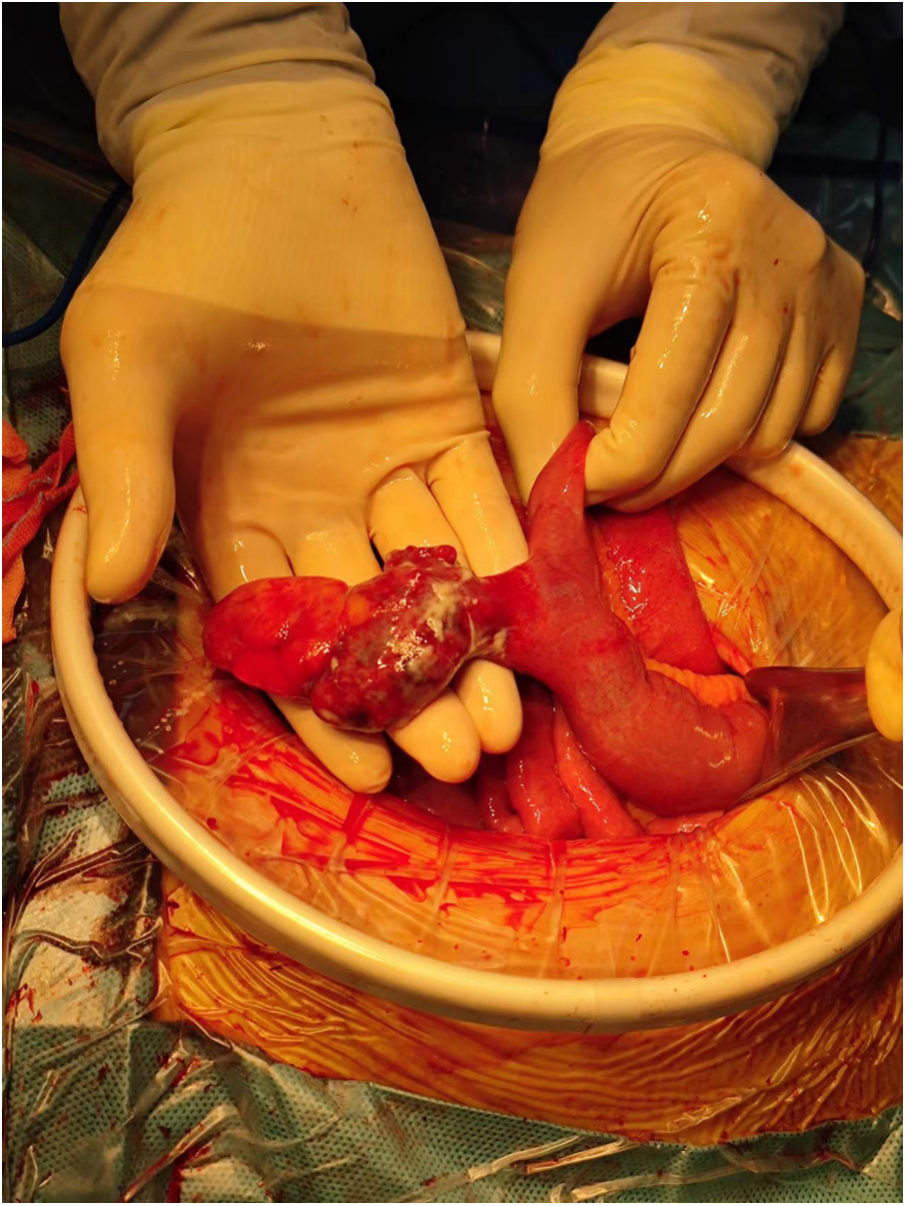
The MD was found during the surgery.

**Figure 4:**
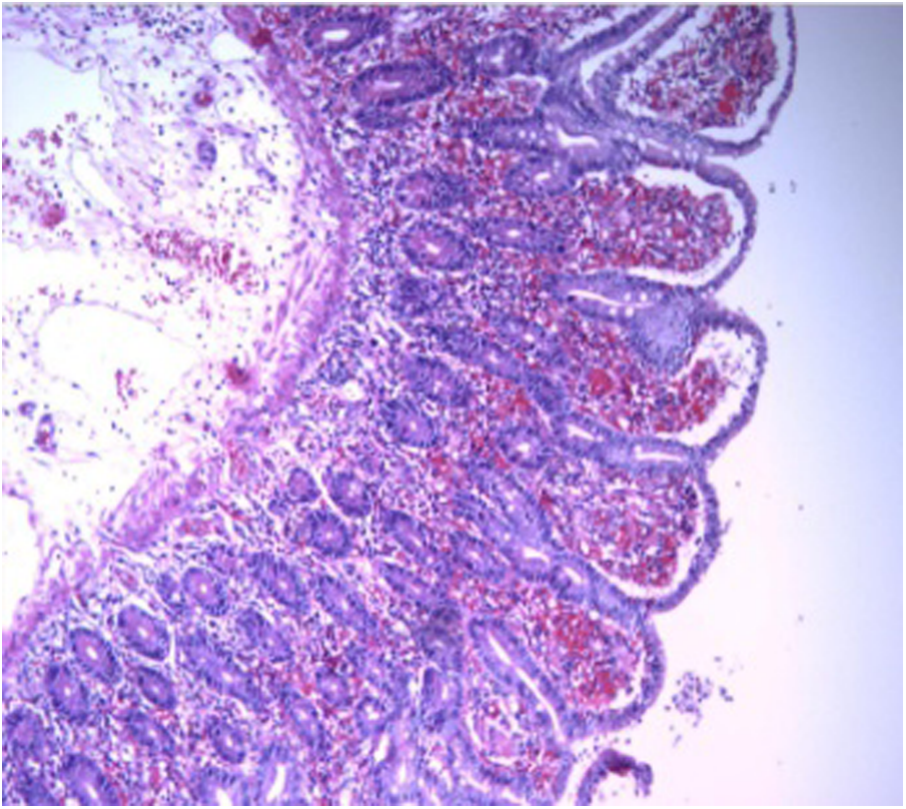
Histopathological findings confirmed a MD.

We performed a caesarean section. The Apgar score at 1, 5, 10, 15 and 20 min was 10, 5 (each item was deducted one point), 9 (reflex irritability was deducted one point), 9 (reflex irritability was deducted one point) and 9 (reflex irritability was deducted one point), respectively. The foetus looked healthy and got full score at 1 min. However, the heart rate, respiratory effort, muscle tone, reflex irritability, and color from the foetus were not the best, so the foetus got 5 points when 5 min. Subsequently, the foetus was rescued by keeping warm and positive pressure ventilation by mask resuscitation bag. And then the foetus got 9 points because the reflex irritability was not the best after rescue. The foetus was finally transferred to the neonatology department. During the operation, the surgeon explored the small intestine MD, with a 360° twist and local necrosis without perforation (Figure 3). The MD was removed and sent for pathological examination, which showed acute inflammatory changes (Figure 4). Finally, the mother and the foetus recovered.

## Discussion

Early diagnosis of acute abdomen in obstetrics is of great significance to improve the life and health of mothers and foetuses. Common obstetrical acute abdomen includes digestive inflammatory diseases, such as appendicitis, acute cholecystitis, acute pancreatitis, and intestinal obstruction. In addition, urinary calculi and torsion of the ovarian cyst pedicle are also seen in the hospital. However, MD is rare. In this study, we reported pregnancy-complicated MD volvulus, which attracted our attention to the diagnosis of obstetrical acute abdomen. When we managed the acute abdomen, we could also consider MD.

MD is the most common anatomic abnormality of the gastrointestinal tract, resulting from the failure of the omphalomesenteric duct to involute during the 5th to 7th weeks of gestation (2) and it has been described as the “rule of twos”: prevalence of 2% in the population, twice as common in men than in women, located 2 feet from the ileocecal valve, and can be 2 inches wide and long (10). MD is usually asymptomatic, and only 2%–4% of people become symptomatic, usually in children (11). A small number of cases can be diagnosed accidentally by an operation or imaging investigation (12). Table 1 illustrates that most cases were diagnosed at emergency surgery. Symptomatic patients with MD present clinical features, such as obstruction of the small bowel, painless bleeding from the rectum, and signs of peritonitis, and common symptoms include vomiting, abdominal pain, fever, and bloody stools (13). Abdominal pain is a very common symptom, whereas nausea and vomiting were also observed. Symptomatic MD during pregnancy is an extremely rare event. There are no more than 40 cases reported so far since 1949, with 22 complicated by perforation (7).

Our patient presented primarily with abdominal pain, which became predominantly left lower abdominal pain, and eventually developed peritonitis. The first case presented with right lower abdominal pain reminiscent of acute appendicitis, with subsequent finding of MD at surgery. In fact, appendicitis could be the most common surgical pathology in the acute abdomen, but some rare diseases should be also considered. MD should not be neglected as one of the differential diagnoses of acute abdominal complaints in pregnancy.

Doctors find it difficult to diagnose MD-complicated pregnancy because it can present unspecific symptoms, such as abdominal pain, nausea, vomiting, and obstipation, which overlap with other conditions and can also occur physiologically during pregnancy. Moreover, moderate leucocytosis can be normal for pregnant women but indicates inflammation in the population without pregnancy, making it more difficult to perform timely evaluations. During pregnancy, symptomatic MD should be distinguished from obstetric diseases such as preterm labour, placental abruption or chorioamnionitis (6).

Ultrasonography is a common radiological investigation in the pregnant patient presenting with an acute abdomen (14). Ultrasonography is not of great importance for adults, but it is of value in paediatrics because it avoids radiation exposure, especially having a higher sensitivity in cases of complications (15). MRI imaging are largely perceived as safe for pregnant patients. It has a higher sensitivity and specificity in the diagnosis of appendicitis in pregnant women (16). However, MRI could not be the first choice because of the duration of scan and resource availability, especially in emergency conditions. Technetium-99 m(99mTc) pertechnetate scintigraphy is regarded as a relatively good diagnostic tool, particularly in children, while lower in adults (17). The scan could be considered if MD is suspected.

CT is common, but doctors can easily overlook MD, which could be considered a small intestinal loop without the presence of complications (18). When MD is accompanied by complications, CT is the best imaging study (19). Magnetic resonance is not of great value in the diagnosis of MD even if complications are present (20). This radionuclide scan has a sensitivity and specificity of 85% and 95% in children, respectively, but its sensitivity and specificity are not as high in adults (10). Ideal diagnosis is by upper GIT contrast follow through or just suspicion. Moreover imaging can be difficult to interpret and US or an abdominal x-ray would have probably been enough. The interpretation of images could be easier by artificial intelligence one day, which brings us more convenience (21). MD is usually diagnosed by exploratory laparoscopy in cases of complications (22).

In our report, CT showed the “whirlpool sign”, indicating small bowel volvulus. Combining CT results and peritonitis, the surgical chief thought it was more likely to be small bowel volvulus and suggested exploratory laparoscopy, by which we found MD complicated by volvulus.

The mainstay of management for symptomatic Meckel's patients is surgical intervention. In the asymptomatic patient, a conservative approach is well justified giving the rarity of complications (23). Every patient in Table 1 presented with abdominal pain and had to undergo surgery. The mode of delivery was either vaginal delivery or cesarean section, which may occur at the time of surgery for the MD, resulting in preterm births requiring neonatal intensive care. For the pregnant with MD, the trimester of pregnancy and foetus mortality should be considered when deciding whether and when to do an operation. The risks and benefits must be weighed against each other.

Many diseases can lead to acute abdomen, while MD is a rare cause of acute abdomen. CT is valuable for the suspicious diagnosis of MD accompanied by symptoms. When managing the acute abdomen, we must not forget the possibility of MD. For the pregnant woman with symptomatic MD, surgery is the preferred mode of management.

## Data Availability

The original contributions presented in the study are included in the article/Supplementary Material, further inquiries can be directed to the corresponding author/s.
